# ﻿A new reef-dwelling coral, *Pavona giannii* sp. nov. (Scleractinia, Agariciidae), with an overview of the skeletal morphology of the type specimens of the genus *Pavona*

**DOI:** 10.3897/zookeys.1260.167263

**Published:** 2025-11-19

**Authors:** Francesca Benzoni

**Affiliations:** 1 Biological and Environmental Science and Engineering Division, Marine Science Program, HaBB Lab, King Abdullah University of Science and Technology, Thuwal, Saudi Arabia King Abdullah University of Science and Technology Thuwal Saudi Arabia

**Keywords:** Morphometrics, museum collections, reef-building coral, skeletal morphology, taxonomy

## Abstract

Hard coral species in the agariciid genus *Pavona* are common in shallow and mesophotic coral reef communities across the Indo-Pacific, but their taxonomy has long been overlooked. *Pavona giannii***sp. nov.** is here described based on newly collected material across the Indian Ocean, and historical museum specimens. In vivo and skeletal morphological features are described, diagnostic characters measured, and comparison with congeners performed. The new species forms an encrusting corallum devoid of raised ridges on its surface. Its corallites are flush with the surface, not inclined, and less than half a corallite diameter apart. Corallites arrangement is thamnasteroid and series can form locally. Where they occur, the radial elements run over the shared walls to the adjacent series’ corallites creating a ladder-like arrangement similar to that observed over the ridges in congeners like *Pavona varians* and *Pavona chiriquiensis*. Despite the lack of ridges, *P. giannii***sp. nov.** has morphological affinities with these two species. However, based on previously published morphometrics and meristics, *P. giannii***sp. nov.** corallites are larger and more crowded, and the primary septa are longer and more numerous. In vivo, the new species is distinguished by fully extended white to beige tentacles during the daytime, giving it a white-bearded appearance. Despite the ecological relevance of *Pavona* corals, a taxonomic revision of the genus is overdue, and the existing molecular studies indicate that it is polyphyletic. Here, the proposed placement of *P. giannii***sp. nov.** in the genus is based on morphological evidence alone and phylogenomic analyses are currently in progress.

## ﻿Introduction

The colonial scleractinian genus *Pavona* Lamarck, 1801 is a common component of the shallow-water coral reef communities across the Indo-Pacific from the Red Sea and the Southwestern Indian Ocean to the East Pacific, spanning tropical and sub-tropical latitudes (Durham and Barnard 1952; Veron and Pichon 1980; Wijsman-Best et al. 1980; Pichon and Benzoni 2007; Glynn et al. 2017; Berumen et al. 2019). *Pavona* corals are zooxanthellate reef-builders actively contributing to the structure and functioning of coral reef ecosystems (Tortolero-Langarica et al. 2022). Some frondose or columnar species can form giant colonies (Mezaki et al. 2014; Siena et al. 2025) and/or monospecific stands locally reaching metric size (Veron 1986; Montagne et al. 2013). *Pavona* species support a diverse and often specialized associated fauna and microbiomes (Hoeksema and van der Meij 2013; Montano and Maggioni 2018; Hu et al. 2020; Mehrotra et al. 2020; Yu et al. 2020; Cheng et al. 2023; Delgadillo Ordonez et al. 2022; van der Schoot and Hoeksema 2024; Bäher et al. 2025). Moreover, some species are depth generalists and thrive below 30 m depth as components of mesophotic coral ecosystems (Luck et al. 2013; Kramer et al. 2019; Longenecker et al. 2019). *Pavona* corals are considered stress-tolerant (Gleason 1993; Huntington et al. 2022) and have been studied for their acclimatory ability in marginal environments and resilience to changes in environmental conditions due to ocean warming (McClanahan, 2000; Solandt et al. 2003; Guzman and Cortés 2007; Tortolero-Langarica et al. 2022; Zhang et al. 2022, 2025; Khen et al. 2024). Despite its ecological relevance and being the target of fundamental and experimental research efforts, species boundaries within the genus *Pavona* and phylogenetic relationships are still only partially explored through a genetic approach. Available molecular data on a subset of species suggest that the genus is polyphyletic and requires formal taxonomic revision (Maté 2003; Moothien Pillay et al. 2006; Kitahara et al. 2012; Luck et al. 2013; Waheed et al. 2015; Terraneo et al. 2017; Quek et al. 2023).

Belonging to the family Agariciidae Gray, 1847, *Pavona* currently includes 20 valid extant species although 57 nominal extant species have been historically ascribed to it (Hoeksema and Cairns 2024). Morphologically, species currently in the genus display remarkable differences in corallum and corallite shape, corallite arrangement, and columellar structure (Veron and Pichon 1980; Latypov 2014). Typically for an agariciid, corallite arrangement in *Pavona* is thamnasteroid: the radial elements run over the corallum surface from a corallite center to the adjacent ones, passing over their walls, which are generally poorly developed and scarcely visible (Chevalier and Beauvais 1987). The formation of new polyps in a colony, and new corallites in its corallum, occurs through intratentacular budding and can continue without the actual loss of organic connection among the buds (Matthai 1948c). This process in some species can lead to the formation of series of polyps aligned in valleys. Adjacent series are separated by variably continuous shared corallite walls that can develop into raised longer ridges or shorter monticules (Matthai 1948a; Veron and Pichon 1980: figs 47–53). These indeed vary in height, length and orientation depending on the species, and on their position in a single colony (Matthai 1948b). The radial elements running above any shape of elongated ridge in *Pavona* are parallel among them and perpendicular to the ridge’s main axis giving a typical ladder-like arrangement (Veron and Pichon 1980: figs 21, 24).

An undescribed species of reef-dwelling *Pavona* was collected at different localities in the Gulf of Tadjoura, Gulf of Aden, Arabian Sea, Strait of Oman, the SW Indian Ocean, and the NE Indian Ocean. It is recognized based on its encrusting growth form, a smooth corallum surface devoid of ridges or monticules, a crowded corallite arrangement, strongly alternating radial elements with the taller ones always flush with the corallum surface giving it an even appearance. In vivo, the species has fully extended polyp tentacles at daytime. Oral disks and/or tentacles are white and give the colonies a distinctive white bearded appearance. The new species skeletal macro- and micromorphology are described and illustrated in detail. Measurements and counts of skeletal characters previously analyzed for two congeners with an encrusting growth form and corallite morphological affinities with it, *Pavona chiriquiensis* Glynn, Maté & Stemann, 2001 and *Pavona varians* (Verrill, 1864) (Maté 2003), were obtained from the undescribed *Pavona* thus allowing a direct quantitative comparison. In the discussion section, type material of nine congeners and reference material from two more is illustrated to show the morphological variation of *Pavona* species and the differences with the new species.

## ﻿Materials and methods

### ﻿Collection and processing

**Figure 1.**
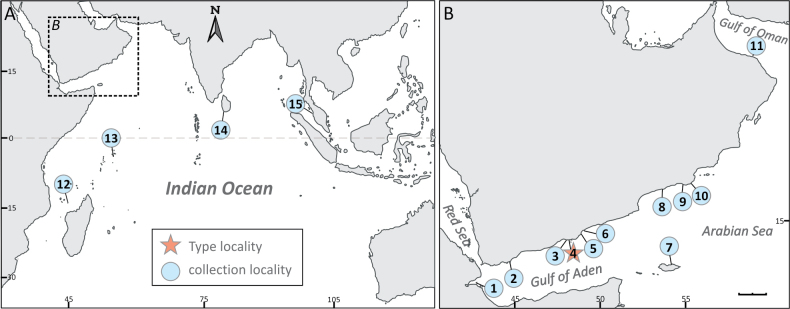
Map showing the type (star) and other collection (circle) localities of *Pavona giannii* sp. nov. **A.** Collection localities in the SW Indian Ocean, and **B.** The seas around the Arabian Peninsula. 1 = Maskali Island, Djibouti; 2 = Aden, Yemen; 3 = Balhaf, Yemen; 4 = Hyllanyia Island, Bir Ali, Yemen; 5 = Burum, Yemen; 6 = Al Mukallah, Yemen; 7 = Hawlaf, Socotra Island, Yemen; 8 = Dhalkut, Oman; 9 = Mirbat, Oman; 10 = Qinqari Bay, Oman; 11 = Muscat, Oman; 12 = Mayotte Island; 13 = Mahé Island, Seychelles; 14 = Galle, Sri Lanka; 15 = Pulau Songsong, Malaysia. Scale bar: 200 km.

Material was collected during SCUBA at 13 localities in the southwestern Indian Ocean (Fig. 1A) and the Gulfs of Tadjoura and Aden, the Arabian Sea, and the Gulf of Oman (Fig. 1B) during multiple expeditions between 2007 and 2022. Before collection, living coral colonies were imaged in situ with digital cameras to document colony growth form, appearance and coloration, and polypar features. Specimens, mostly colony fragments, were sampled by hammer and chisel. Once on land, each specimen was tagged and subsampled to preserve coral tissue in molecular grade ethanol for DNA extraction. Coralla were then left overnight in a sodium hypochlorite solution (household bleach) to remove organic matter, rinsed in freshwater, and dried.

### ﻿Imaging and morphological analysis

For macro-morphological observations of corallum surface and corallite arrangement, specimens were photographed with a Nikon Coolpix digital camera with a reference scale and through a Leica M80 microscope equipped with a Leica IC80HD camera at known magnification. To investigate and describe the skeletal features micro-morphology and their variability, fragments were obtained from two distinct specimens displaying different degrees of calcification of the corallum, namely UNIMIB BAL253 (thinner) and UNIMIB AD016 (thicker). Fragments were grinded, mounted on stubs using silver glue, sputter-coated with conductive gold film and examined using a Vega Tescan Scanning Electron Microscope at University of Milano-Bicocca, Italy. For specimen size measurements, a caliper was used. Corallite and radial element measurements and counts were taken on digital images with visible reference scale using Image J v. 1.54p (Schneider et al. 2012) for 13 specimens. For each specimen, five corallites were selected for measurements and counts. Only full-grown corallites, not adjacent and not undergoing budding were considered. Following Maté (2003: fig. 3), the following skeletal characters that provided statistically significant differences among *Pavona* species were measured and counted: maximum calicular diameter, minimum calicular diameter, maximum columellar diameter, minimum columellar diameter, main septa (S1, 2) length, number of septa, number of septa reaching the columella. Statistical analysis to evaluate if any of the characters were statistically significantly different in the new species compared to the encrusting species examined by Maté (2003) was not possible because the original dataset was not included in which only averages (± SE) were reported for each character. Therefore, comparison of characters is hereafter performed based on the available information.

### ﻿Repositories and institutional acronyms

The specimens collected for this study are deposited in the following repositories: Muséum national d'Histoire naturelle (**MNHN**), Paris, France; Florida Museum of Natural History (**UF**), Gainesville, Florida, USA; King Abdullah University of Science and Technology (**KAUST**), Thuwal, Saudi Arabia; University of Milano-Bicocca (**UNIMIB**), Milan, Italy; University of the Seychelles (**UNISEY**), Anse Royale, Mahé, Seychelles.

In September 2004, a visit to the Natural History Museum (**NHMUK**), London, UK, allowed the discovery of historical specimens showing the same diagnostic macro and micro-morphological characters as the collected material for this study. In the accompanying labels, they had all been identified as *Pavona explanulata* (Lamarck, 1816). Collected in the last century by different scientists from the Seychelles, Sri Lanka, and Malaysia, these specimens extend the known geographic distribution of the new species to two additional localities in the NE Indian Ocean (Fig. 1A).

### ﻿Examined type and reference material

For comparative purpose, type or representative specimens of *Pavona* species examined or collected by the author, respectively, were included in this study and are listed hereafter. The holotype of *Pavona diminuta* Veron, 1990 (QMT G32480) was examined and photographed at the Queensland Museum Tropics (**QMT**), Townsville, Queensland, Australia, in May 2009. The holotype of *Pavona minuta* Wells, 1954 (USNM 44786) was studied and photographed in June 2009 at the United States National Museum (**USNM**), Washington DC, USA. During a subsequent visit in March 2023, the type specimens of *Pavona decussata* Dana, 1846 (USNM 201) and *Pavona chiriquiensis* Glynn, Maté & Stemann, 2001 (USNM 100871) were also examined and imaged. Images of the holotypes of *Pavona diffluens* (Lamarck, 1816) (MNHN IK-210-587) and *Pavona maldivensis* (Gardiner, 1905) (NHMUK 1937.11.17.806) were taken at the MNHN in February 2014 and at the NHMUK in September 2024, respectively. In October 2024, the type series of *Pavona varians* Verrill, 1864, a paratype of *P. chiriquiensis* (YPM 24153), and syntypes of *Pavona clavus* (Dana, 1846) (YPM 6249) and *Pavona gigantea* (Verrill, 1869) (YPM 1679A) were studied and imaged at the Yale Peabody Museum (**YPM**). An image with scale of specimen YPM IZ.001806.CN (syntype) was kindly provided by E. Lazo-Wasem. The type specimens of *Pavona cactus* (Forskål, 1775), type locality Red Sea, and *Pavona explanulata* (Lamarck, 1816), type locality Indian Ocean, were not examined. Instead, specimens KAUST SA 205, matching the original description of *P. cactus*, and KAUST SA 231, *P. explanulata*, from the Farasan Banks, Saudi Arabian Red Sea, were imaged at the Red Sea Research Center (RSRC), KAUST after collection in 2013.

## ﻿Taxonomic account

**Family Agariciidae Gray, 1847**

**Genus *Pavona* Lamarck, 1801**

### ﻿ Pavona giannii sp. nov.

Taxon classificationAnimaliaScleractiniaAgariciidae

C10E3199-82B0-59A0-B3FF-1BEB51595C49

https://zoobank.org/8396733D-1C70-4F02-BD5C-655C050C3C98

**Figure 2.**
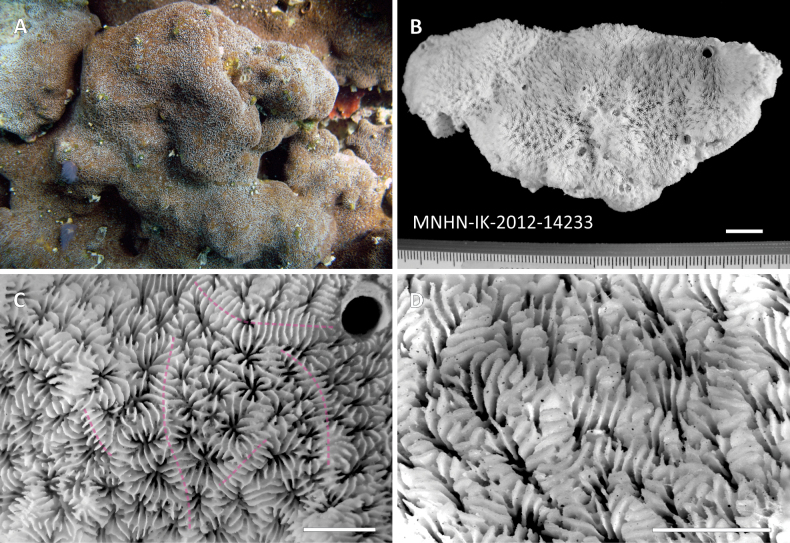
Holotype of *Pavona giannii* sp. nov. MNHN-IK-2012-14233 **A.** the whole colony in situ at Hyllanyia Island, Bir Ali, Yemen, prior to sampling and **B.** corallum of the sampled fragment. **C.** Top view of the corallum showing corallite arrangement and the position of some of the corallite series boundaries (dashed pink lines) marked by the presence of parallel radial elements running over and across them, and **D.** side view showing its even surface given by equally high and flat top margins of S1 and S2 septa, and the deep-seated fossae. Scale bars: 1 cm (**B**); 5 mm (**C, D**).

**Figure 3.**
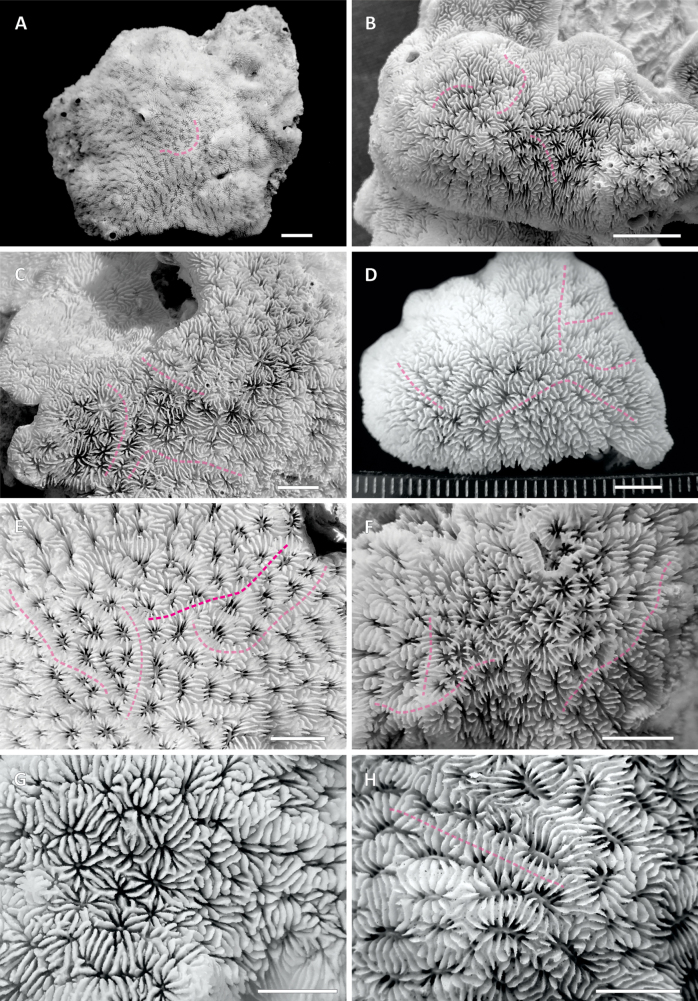
Top views of the coralla showing corallite arrangement in the *Pavona giannii* sp. nov. specimens collected for this study: **A.**UNIMIB AD016, from Aden, Yemen; **B.**UNIMIB MU128, from Al Mukallah, Yemen; **C.**UNIMIB BA066, from Bir Ali, Yemen; **D.**UNIMIB SO078, from Socotra Island, Yemen; **E.**UNIMIB MY069, from Mayotte Island; **F.**UF 18088 (collection code OM226), from Mirbat, Oman; **G.**UF 18089 (collection code OM722), from Muscat, Oman; **H.** Same specimen as in **A.** Dashed pink lines indicate the position of some of the corallite series boundaries marked by the presence of parallel radial elements running over and across them. Scale bars: 1 cm (**A, B**); 5 mm (**C–H**).

Figs 2, 3, , 5, 6, 7

#### Type locality.

**Yemen**: Shabwa Province, Bir Ali, Hyllanyia Island, 13°59.183'N, 48°19.137'E; depth 5 m; 11 November 2008, F. Benzoni leg.

#### Type material.

***Holotype*** • 1 colony fragment (10.5 x 5.1 cm, Fig. 2); Original label: “Bir Ali, Hyllanyia Island, Yemen; 13°59.183'N, 48°19.137'E; 11 Nov. 2008; F. Benzoni leg.; UNIMIB-Creocean-Total Yemen Coral Biodiversity exped.; collection code BA034; MNHN-IK-2012-14233”.

#### Other material.

**Figure 4.**
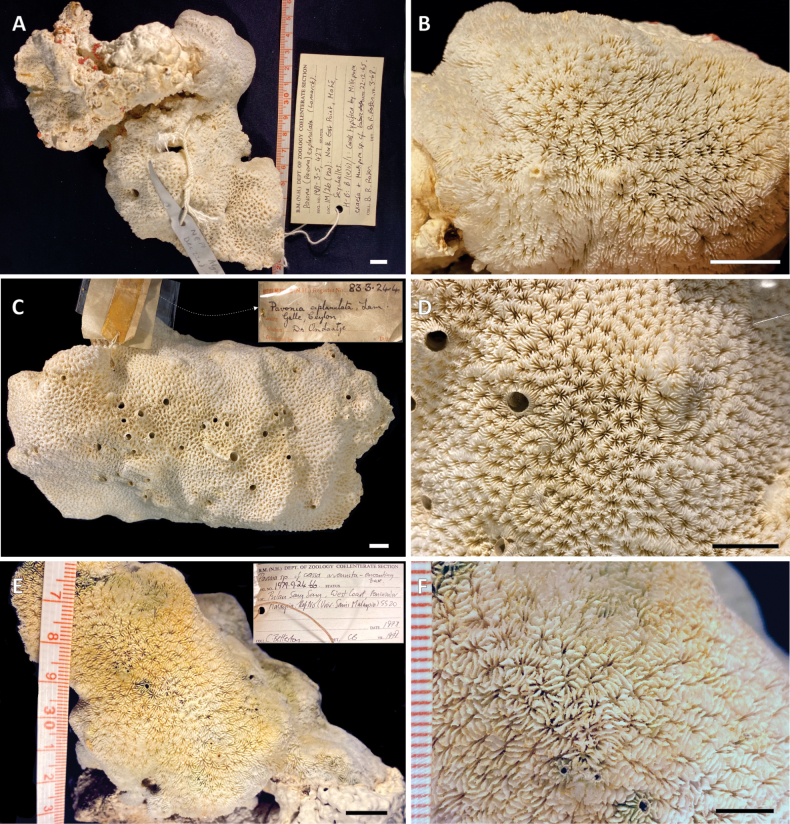
Corallum and corallite macro-morphology of the Natural History Museum (UK) specimens originally identified as *Pavona explanulata* and here re-identified as *Pavona giannii* sp. nov. from localities 13–15 in Figure 1. **A.** View of the whole corallum of specimen NHMUK 1981.3.5.427 from Mahé Island, Seychelles, encrusting a large piece of coral rubble also colonized by other benthic organisms including crustose coralline algae (CCA) and benthic foraminifera; **B.** Detail of **A** showing corallite arrangement; **C.** Top view of specimen NHMUK 83.3.24.4 from Galle, Sri Lanka; **D.** Detail of the corallum surface of the specimen in **C; E.** Top view of specimen NHMUK 1979.9.24.66 from Pulau Songsong, Malaysia, encrusting a block of coral rubble also overgrown by CCA (bottom right); **F.** Closer view of the same specimen in **E.** Scale bars: 1 cm (**A–E**); 5 mm (**F**).

**Figure 5.**
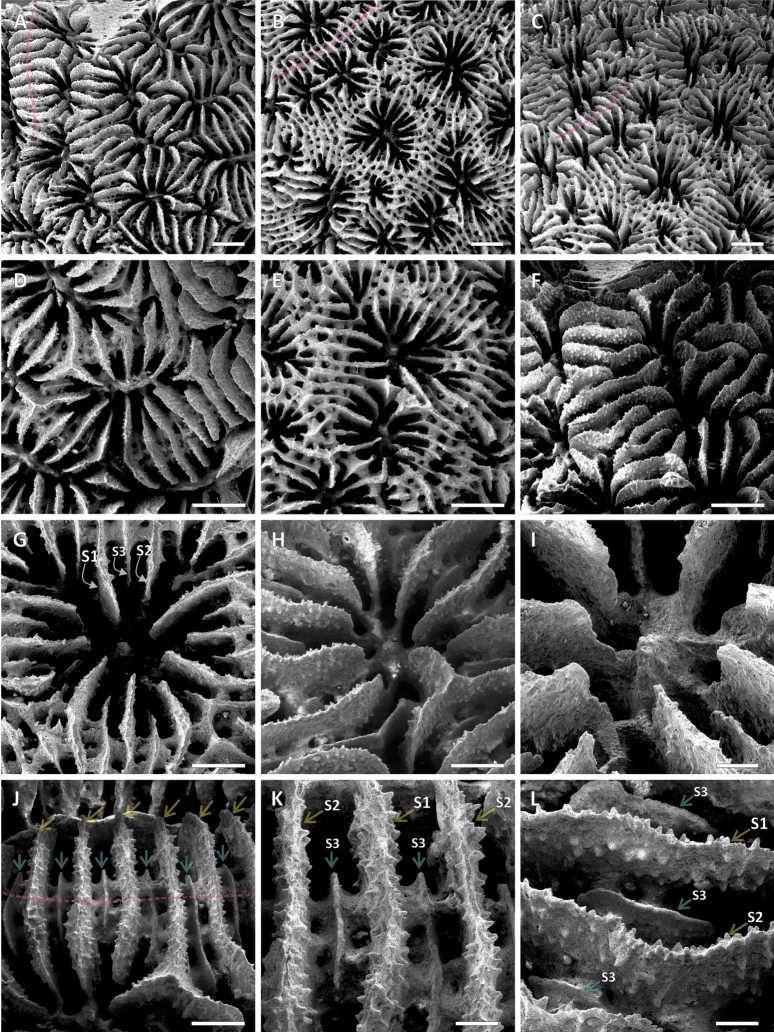
Scanning Electron Microscopy of the skeletal micromorphology of *Pavona giannii* sp. nov. **A.** Top view of a well-calcified specimen showing crowded corallite arrangement and thick radial elements masking the underlying skeletal structures. **B.** Same view in a less calcified specimen showing thinner radial elements connected by visible synapticulae and a corallite series at the top left corner (the position of the series boundary marked by the dashed pink line). **C.** Side view of **B** showing the flattened corallum surface and the flush top margins of the S1–2 radial elements. **D.** Closeup of one of the corallites of the specimen in **A. E.** Closeup of the corallites of the specimen in **B. F.** Side view of the specimen in **A** showing the flattened and flush top margins of the S1–2 going from one corallite center to the adjacent ones. **G.** Top view of a corallite where the three radial elements orders (S1>S2>S3) are indicated by the arrows. **H.** Side view of a laterally flattened columella sitting deep in a corallite fossa. **I.** Inner septal margins fusing with the columella. **J.** Top view of the parallel radial elements running over the synapticular corallite walls, perpendicularly to the series boundary direction (pink dashed line), the thicker S1–2 are indicated by the transparent yellow arrows and the thinner S3 by the transparent turquoise arrows. **K.** Close up of **J** showing the difference in lateral side ornamentation between the thicker S1–2 and the thinner S3. **L.** Side view of the radial elements in **K** showing the difference in height between the taller S1–2 and the shorter S3. **A, F, H, I–L** = UNIMIB AD016; **B, C, E, G** = UNIMIB BAL253. Scale bars: 1 mm (**A–F**); 500 mm (**G, H, J**); 200 mm (**I, K, L**).

**Figure 6.**
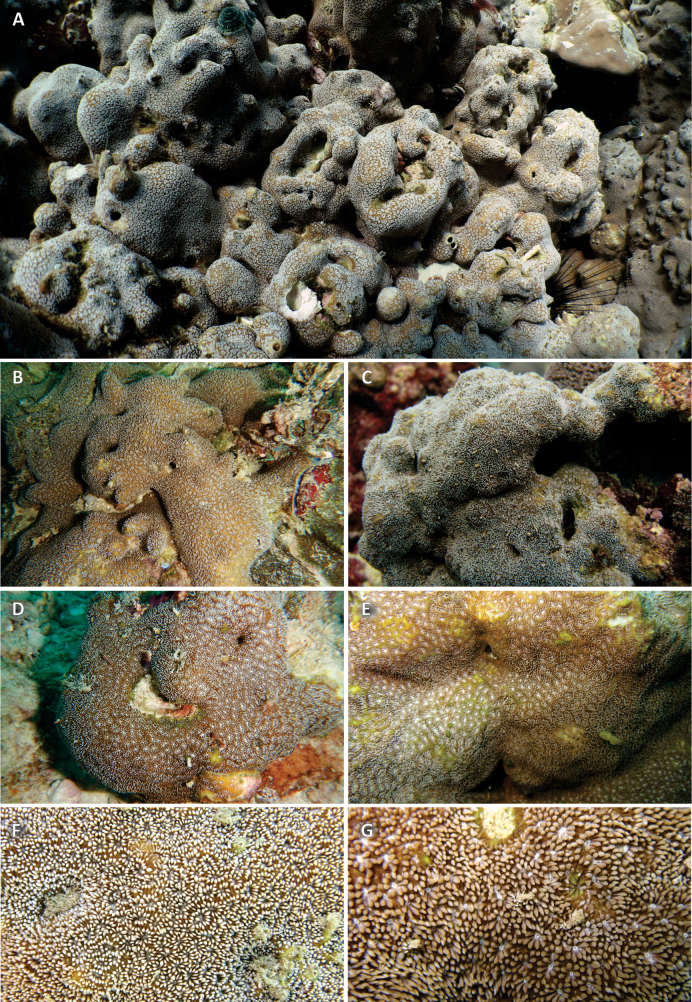
*Pavona giannii* sp. nov. in situ. **A.** Typically white extended tentacles in the colony, from which specimen UNIMIB BAL252 was collected, encrusting a submassive *Porites* (living portion at the top right-hand side) at Balhaf, Yemen; **B.** Colony of UF 17957, Mirbat, Oman; **C.** Fully extended white tentacles covering the whole surface of the colony of UNIMIB SO078 at Socotra Island, Yemen; **D.** Colony with partially retracted tentacles allowing the polyps’ white oral disks to show, Mahé Island, Seychelles; **E.** Colony of UNIMIB MY069 at Mayotte Island; **F.** Colony with white tentacles and grey oral disks Balhaf, Yemen; **G.** Close-up of the polyps of colony UNIMIB BU049 with beige tentacles and white oral disks at Burum, Yemen.

**Figure 7.**
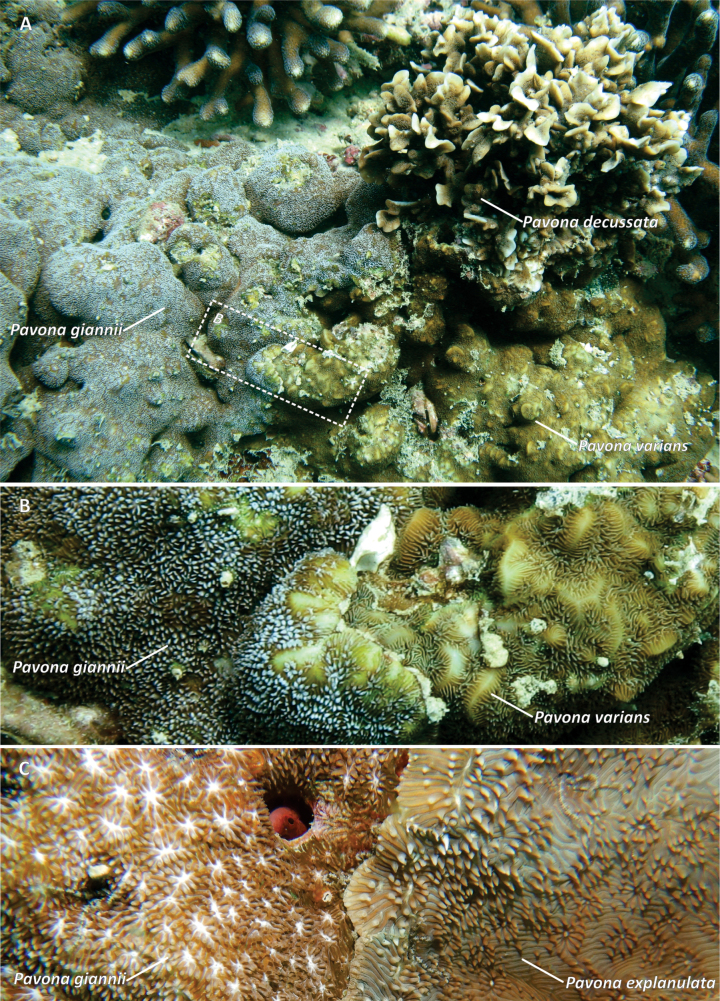
*Pavona giannii* sp. nov. and congeners side-by-side in situ allowing a direct comparison of the polyp macro-morphology and coloration. **A.***P. giannii* sp. nov., *Pavona decussata* and *Pavona varians* at Bir Ali, Yemen;. **B.** Detail of A showing a margin of the *P. giannii* sp. nov. colony, devoid of ridges and with the typical white tentacles, actively growing over the *P. varians* one with well-developed ridges and shorter brown tentacles; **C.** Margin of a *P. giannii* sp. nov. with crowded polyps, light brown tentacles and white oral disks, a less commonly observed color variant, being overgrown by *Pavona explanulata* with extended brown tentacles and well-distanced polyps. Dashed polygon in **A** shows the portion of the image shown in **B.**

**Djibouti** • 1 colony fragment (Fig. 7C, part of the colony in situ); Maskali Island; 11°42.38'N, 43°9.24'E; 29 Feb. 2020; F. Benzoni leg.; Dolphin Cruise exped.; KAUST DJ403. **Yemen** • 1 colony fragment (Figs 3A, H, 5A, D, F, H–L); Aden, Ras Antouk; 12°45.085'N, 45°1.659'E; 8 Mar. 2009; F. Benzoni and M. Pichon leg.; UNIMIB-Creocean-Total Yemen Coral Biodiversity exped.; UNIMIB AD016 • 1 colony fragment (Fig. 6A, whole colony in situ); Balhaf; 13°58.402'N, 48°11.549'E; 22 Mar. 2014; F. Benzoni leg.; Creocean-Total Balhaf LNG Plant monitoring program exped.; UNIMIB BAL252 • 1 colony fragment (Fig. 5B, C, E, G); Balhaf; 13°58.413'N, 48°10.532'E; 24 Mar. 2014; F. Benzoni leg.; Creocean-Total Balhaf LNG Plant monitoring program exped.; UNIMIB BAL253 • 1 colony fragment; Bir Ali; 13°59.116'N, 48°15.372'E; 16 Nov. 2008; F. Benzoni leg.; UNIMIB-Creocean-Total Yemen Coral Biodiversity exped.; UNIMIB BA010 • 1 colony fragment; Bir Ali; 13°59.094'N, 48°14.018'E; 16 Nov. 2008; F. Benzoni leg.; UNIMIB-Creocean-Total Yemen Coral Biodiversity exped.; UNIMIB BA017 • 2 colony fragments (Fig. 3C); Bir Ali; 13°59.180'N, 48°15.692'E; 19 Nov. 2008; F. Benzoni leg.; UNIMIB-Creocean-Total Yemen Coral Biodiversity exped.; UNIMIB BA066 • 2 colony fragments (Fig. 6G, colony in situ); Burum; 14°19.266'N, 48°59.641'E; 18 Mar. 2009; F. Benzoni and M. Pichon leg.; UNIMIB-Creocean-Total Yemen Coral Biodiversity exped.; UNIMIB BU049 • 2 colony fragments; Al Mukallah; 14°30.923'N, 49°9.254'E; 17 Mar. 2007; F. Benzoni and M. Pichon leg.; UNIMIB-Creocean-Total Yemen Coral Biodiversity exped.; UNIMIB MU085 • 1 colony (Fig. 3B); Al Mukallah; 14°30.696'N, 49°9.360'E; 18 Mar. 2007; F. Benzoni and M. Pichon leg.; UNIMIB-Creocean-Total Yemen Coral Biodiversity exped.; UNIMIB MU128 • 1 colony fragment (Figs 3D, 6C, colony in situ); Socotra Island, Hawlaf; 12°40.662'N, 54°4.497'E; 14 Mar. 2010; F. Benzoni and M. Pichon leg.; UNIMIB-Creocean-Total Yemen Coral Biodiversity exped.; UNIMIB SO078. **Oman** • 1 colony fragment (10 x 8 cm); Dhalkut; 16°41.235'N, 53°11.749'E; depth 9.4 m; 4 Dec. 2022; F. Benzoni leg.; Oman Bioblitz exped.; collection code OM0895; UF 17903 • 2 colony fragments (Fig. 6B, colony in situ); Mirbat, Eagles Bay; 16°56.377'N, 054°47.799'E; depth 5.4 m; 9 Jan. 2022; F. Benzoni leg.; Oman Bioblitz exped.; collection code OM0096; UF 17957 • 1 colony fragment (Fig. 3F); Mirbat, Qinqari Bay; 17°0.561'N, 55°1.240'E; depth 6 m; 12 Jan. 2022; F. Benzoni leg.; Oman Bioblitz exped.; collection code OM0226; UF 18088 • 1 colony fragment (Fig. 3G); Muscat, Jazirat Al Fahl; 23°40.953'N, 58°30.011'E; depth 5.9 m; 1 Feb. 2022; F. Benzoni leg.; Oman Bioblitz exped.; collection code OM0722; UF 18089 • 1 colony fragment; Mirbat, Marriott Wreck; 16°56.933'N, 054°43.686'E; depth 10.4 m; 7 Jan. 2022; F. Benzoni leg.; Oman Bioblitz exped.; collection code OM0017; UF 18090. **Mayotte** • 3 colony fragments (Figs 3E, 5E, part of the colony in situ); Îlot Mtsamboro; 12°38.031'S, 45°1.140'E; 1 Jun. 2010; F. Benzoni leg.; Tara Oceans exped.; UNIMIB MY069. **Seychelles** • 1 colony fragment; Mahé Island, Horse Shoe Reef; 24 Feb. 2019; F. Benzoni and R. Arrigoni leg.; University of Seychelles Outer Islands coral collection facility exped.; UNISEY SY079 • 1 colony fragment; same data as for preceding; UNISEY SY080 • 1 colony; Mahé Island, Le Cap; 12 Jan. 1966; B.R. Rosen leg.; NHMUK 1981.3.5.425 • 1 colony; Mahé Island, Baie Ternay; 28 Dec. 1965; B.R. Rosen leg.; NHMUK 1981.3.5.426 • 1 colony (Fig. 4A, B); Mahé Island, North East Point; 22 Dec. 1965; B.R. Rosen leg.; NHMUK 1981.3.5.427. **Sri Lanka** • 1 colony (Fig. 4C, D); Galle; W.C. Ondaatje leg.; NHMUK 1883.3.24.4 • 1 colony; W.C. Ondaatje leg.; NHMUK 1883.5.23.8. **Malaysia** • 1 colony (Fig. 4E, F); Pulau Songsong, West Coast, Peninsular Malaysia; C. Betterton leg.; NHMUK 1979.9.24.66.

#### Description.

Colonial, corallum encrusting with attached margins, growing on the underlying substrate and broadly following its surface relief (Figs 2–4, 6, 7); maximum observed thickness 1 cm. Corallum surface smooth and devoid of ridges, crests, monticules or hydnophorae (Figs 2B–D, 3, 4). Corallites crowded, less than a corallite diameter apart, and thamnasteroid in arrangement (Figs 2B–D, 3, 4, 5A–F). Budding intratentacular, locally leading to the formation of variably developed corallites series by repeated incomplete separation of walls after budding. Their outline is variable even within the same colony ranging from elliptical to kidney-shaped (Fig. 3H), polygonal (Fig. 3F) or irregular (Figs 2C, 3C). Corallites 2.4 mm (± 0.1 SE) in average maximum diameter and 1.7 mm (± 0.1 SE) in average minimum diameter. On average, 23 septa (± 1 SE) occur per corallite (Table 1), the 6 S1 reaching the columella, their inner margin fusing with it; their average length is 0.9 mm (Table 1). Septa arranged in three orders (Fig. 5G, K, L), those of the first and second (S1 and S2, respectively) sub-equal in thickness and length, all attaining the same height (Fig. 5J–L). S2 may reach the columella or remain slightly shorter either with free margin or occasionally fusing with the columella more deeply in the fossa. Third order septa (S3) are always present, almost complete. S3 septa are less than ½ of S1 and S2 in length (Fig. 5J, K), never reaching the columella, and are thinner and shorter in height than S1–2 (Fig. 5L). Septal sides of S1 and S2 ornamented with scattered blunt granules, S3 sides smooth (Fig. 5J–L). Synapticulae connecting radial element lateral sides horizontally can be visible in less densely calcified specimens (Fig. 5B, C, E). S1 and S2 upper margins flush, flattened and running parallel to corallum surface giving it an overall even appearance (Figs 2D, 5C, F). Where corallite series occur, radial elements run beyond the series shared wall to the adjacent series, perpendicularly to its axis (transparent pink dashed lines in Figs 2C, 3A–F, H, 5A–C, J). Above the shared wall, they are mostly parallel, their upper margin flattened and flush with the corallum surface (Fig. 5C). Viewed from above, the radial elements running over the adjacent corallite series shared wall resemble frets over a guitar fingerboard (Fig. 5J), their ladder-like arrangement identical to that usually observed over the ridges forming in other congeners (Veron and Pichon 1980: figs 21, 24). Columella present, solid and made of a single blunt process its tip sitting lower than the upper septal margin (Fig. 5D–I). Maximum and minimum average columella diameter 0.5 and 0.2 mm, respectively (Table 1). Columella transverse section below the tip shape ranging from circular to elongated and dash-like in outline.

**Table 1.** Comparison of mean (±SE) corallite and radial elements dimensions (mm) and counts among *Pavona giannii* sp. nov., *Pavona chiriquiensis*, and *Pavona varians*. Character names, measurements and counts for *P. chiriquiensis* and *P. varians* are derived from Maté (2003: table 6) retaining the original number of decimal places used therein.

	P. giannii sp. nov.	P. chiriquiensis	P. varians
	Mean (±SE)	Mean (±SE)	Mean (±SE)
Maximum calicular diameter	2.39 (±0.09)	1.85 (±0.05)	1.31 (±0.03)
Minimum calicular diameter	1.66 (±0.10)	1.11 (±0.04)	1.50 (±0.02)
Maximum columellar diameter	0.46 (±0.05)	0.34 (±0.03)	0.26 (±0.01)
Minimum columellar diameter	0.20 (±0.02)	0.20 (±0.02)	0.16 (±0.01)
Main septa length	0.90 (±0.06)	0.62 (±0.02)	0.47 (±0.01)
Number of septa	23.35 (±0.74)	20.02 (±0.53)	19.86 (±0.62)
Number of septa reaching the columella	6.34 (±0.31)	7.17 (±0.16)	7.88 (±0.15)

Typically, polyp tentacles are extended in the daytime; therefore, coenosarc, tentacle, oral disc, and mouth coloration is usually visible in situ. Living tissue surrounding the mouth orifice and the oral disc is white (Figs 6D–G, 7) to light grey (Fig. 2A). Tentacles commonly white, giving colony surface a bearded appearance (Figs 2A, 6A–F, 7). Occasionally, tentacles can also be light green or brown (Fig. 6A), seldom brown (Fig. 7C). The coenosarc is typically brown (Figs 2A, 6A, C, D, F, G, 7A, B) to savora mustard yellow (Fig. 6B, E).

#### Etymology.

This species is named after Giambattista J.d.C. Benzoni, known as Gianni (1946-2024) for his unconditional support throughout my personal and professional life, and the graceful pride he took in being a coral taxonomist’s father.

#### Distribution and habitat.

*Pavona giannii* sp. nov. is a reef-dwelling species known from multiple localities north and south of the equator in the tropical Indian Ocean (Fig. 1). This species can be part of shallow water coral reef communities occurring in well-lit conditions between 1 and 15 m depth. It can encrust blocks of coral rubble, dead coral colonies or limestone, and can also grow on non-carbonate bedrock as observed in the Gulf of Aden and Arabian Sea. There, *Pavona giannii* sp. nov. seasonally withstands the low temperature and nutrient rich waters brought by the summer Arabian Sea upwelling (Sheppard and Sheppard 1991). These recurrent conditions limit coral reef formation and select the scleractinian taxa able to withstand a pseudo-high latitude effect (Benzoni et al. 2003, 2012).

## ﻿Discussion

**Figure 8.**
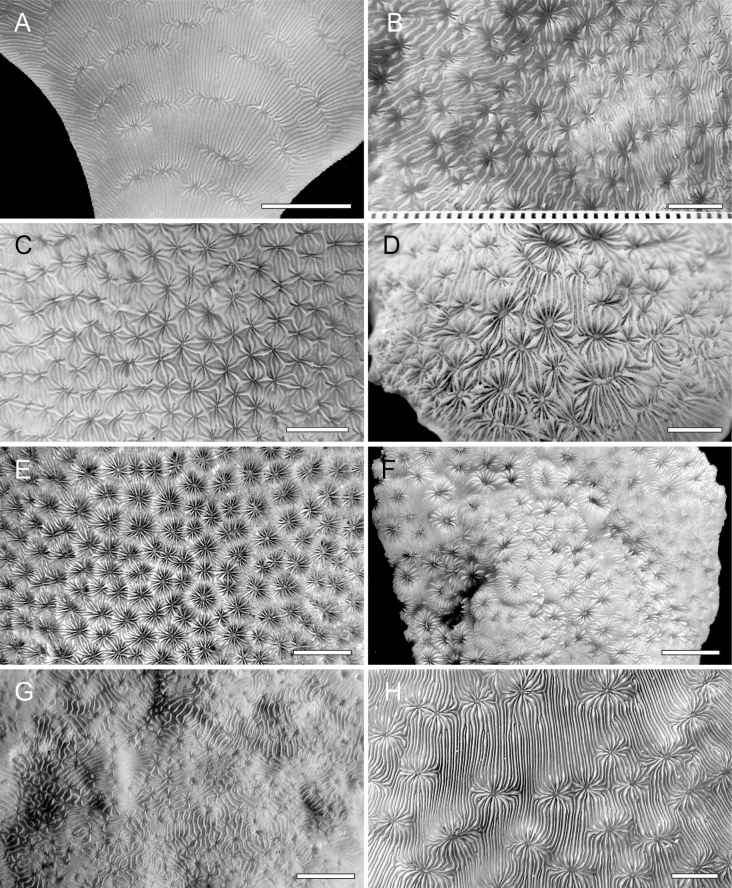
Corallum and corallite morphology of type and collected specimens of *Pavona* species. **A.** The genus type species *Pavona cactus* (KAUST SA205), Farasan Banks, Red Sea (type locality); **B.***Pavona gigantea* syntype (YPM 1679A); **C.***Pavona diminuta* holotype (QMT G 32480); **D.***Pavona diffluens* holotype (MNHN-IK-210-587); **E.***Pavona clavus* syntype (YPM 6249); **F.***Pavona maldivensis* holotype (NHMUK 1937.11.17.806); **G.***Pavona minuta* holotype (USNM44786); **H.***Pavona explanulata* (KAUST SA231), Farasan Banks, Red Sea. Scale bars: 5 mm.

The morphological differences in terms of corallum and corallite morphology are so pronounced among the species currently ascribed to the scleractinian genus *Pavona* (Fig. 8) that previous authors have expressed perplexities concerning its diagnostic morphological characters and questioned its boundaries with the closely related genera *Leptoseris* Milne Edwards & Haime, 1849 and *Gardineroseris* Scheer & Pillai, 1974 (Veron and Pichon 1980; Latypov 2014). Indeed, molecular analyses have shown that the genus as currently conceived is polyphyletic. However, so far, phylogenetic relationships among species ascribed to *Pavona* could only be partially resolved due to reduced species sampling and/or poorly informative genetic and/or morphological data in available studies (Maté 2003; Moothien Pillay et al. 2006; Luck et al. 2013; Terraneo et al. 2017; Quek et al. 2023). Therefore, an integrated systematics approach to the genus revision, akin to that adopted for other genera (e.g. Arrigoni et al. 2020), remains unattainable until more specimens of the currently valid species from multiple localities, including topotypes, are examined in a phylogenomic study.

*Pavona giannii* sp. nov. presents a unique combination of morphological characters that distinguish it from its congeners (Table 2, Fig. 8). Based on corallum morphology, *P. giannii* sp. nov. typically forms encrusting coralla with attached margins and is readily distinguished from the massive and submassive *Pavona gigantea* (Verrill, 1869), *Pavona duerdeni* Vaughan, 1907, *Pavona diminuta* Veron, 1990, *Pavona bipartita* Nemenzo, 1979, and *Pavona diffluens* (Lamarck, 1816), the two latter species also featuring free corallum margins. At the corallite level, the new species can form series, is devoid of ridges, its corallites are closer than 1 corallite apart, corallite wall is flush with the corallum surface, corallite diameter is larger than 2 mm but smaller than 3 mm, septa thickness is unequal, the septal upper margin above the corallite wall is flattened, and the columella is sitting low in the fossa (Table 2). Therefore, *P. giannii* sp. nov. is different from: *P. gigantea*, which has corallites equal or larger than 4 mm in diameter and one or more corallite diameters apart (Fig. 8B); *P. duerdeni* with corallites smaller than 2 mm in diameter and equal septal thickness (Vaughan 1907: fig2a); *P. diminuta*, which seldom forms corallite series and has a high columella (Fig. 8C); *Pavona bipartita* with rounded septal upper margin above the corallite wall and a high columella; and from *P. diffluens* with corallites more than 4 mm in diameter and an obvious and high columella (Fig. 8D). Among massive *Pavona* species, *Pavona giannii* sp. nov. shares some similarities at the corallite level with *Pavona distincta* Latypov, 2014. Based on the species original description and the available image of part of the type specimen surface, both species form a smooth corallum surface with crowded corallites, septal length is S1>S2>S3, and upper radial element margins are flush. However, according to Latypov (2014: 389) *P. distincta* forms massive colonies, “*all septa are of an equal height and thickness, massive, even, straight, and weakly ornamented with a few tubercles*”, a columella is absent, and corallites are not arranged in series. Instead, in *P. giannii* sp. nov. colonies are encrusting, S1 and S2 are taller and thicker than S3 (Fig. 5J–L), the lateral side ornamentation for S1 and S2 is well-developed (Fig. 5H–L), a columella is present (Fig. 5D, E, G–I), albeit sitting sometimes deep in the fossa, and corallites can be arranged in series (Figs 2, 3). Another predominantly massive *Pavona* species that can also form encrusting colonies is *Pavona venosa* (Ehrenberg, 1834). This species is characterized by tall and acute corallite series walls forming continuous ridges, and septal upper margin plunging into the fossa (Veron and Pichon 1980: figs 55‒58), and therefore readily distinct from the smooth *P. giannii* sp. nov. corallum surface.

**Table 2.** List of the corallum and corallite characters and their states for *Pavona giannii* sp. nov. and the currently valid *Pavona* species. Corallum growth form (CGF): massive (mv), submassive (sm), columnar (cl), frondose (fr), foliose (fl), encrusting (en); Corallite arrangement in series (CS): mostly present (yes), partially present, or only in some coralla but not in others (pars), mostly absent (no); Raised corallum ridges (CR) present (yes), absent (no); short (sh), long (lo); Corallite distance (CDIS): more than 1 corallite diameter apart (>), less than 1 corallite diameter apart (<); Corallite wall vertical development (CW): raised from the corallum surface (r), flush with the corallum surface (f); Corallite diameter (CD): CD < 2 mm (a); 2mm ≤ CD <4 mm (b), CD ≥4 mm (c); corallite outline (CO): circular (ci), elliptical (el), polygonal (po), irregular (ir), indistinct (in); Septal thickness (ST): equal or sub-equal (eq), unequal (un); Septal upper margin shape above the corallite wall (SUM): rounded (ro), flattened (fl); Columella vertical development in the fossa respective to the septal upper margin (CDE): high (hi), low (lo), indistinct (in). * = corallum margins free, not attached to the substrate. na = information not available from the original description and illustration, type not examined. ^ = from the subsequent description by Milne-Edwards (1860: 67).

Pavona species	CGF	CS	CR	CDIS	CW	CD	CO	ST	SUM	CDE
*P. cactus*	fr	yes	yes	>	f	a	el-in	eq	fl	lo
*P. danai*	fr	yes	yes	>	f	a	ci-el-in	un	ro	hi
*P. frondifera*^	fr	yes	yes	na	f	a	na	un	na	hi
*P. decussata*	fr	yes	yes	<	f	b	el-po-in	un	ro-fl	lo
*P. divaricata*	fr	yes	yes	</>	f	b	el-in	eq	ro-fl	hi
*P. minor*	fr	yes	yes	na	f	a	po-in	na	ro	in
*P. diminuta*	mv	pars	no	<	f	b	el-in	un	fl	hi
*P. distincta*	mv*	no	no	<	f	na	in	eq	fl	in
*P. gigantea*	mv	pars	no	>	f	c	ci-el	un	ro	hi
*P. duerdeni*	mv	pars	no	<	f	a	ci-el	eq	ro	lo
*P. diffluens*	sm-c-en*	no	no	<	r-f	c	ci-el	un	ro	hi
*P. clavus*	sm-c	pars	no	<	f	b	ci-el-ir	un	ro	hi
*P. bipartita*	sm-en*	pars	no	<	f	b	ci-el-ir	un	ro	hi
*P. venosa*	m-sm-en	pars	yes	<	r	b	po-ir	eq	ro	lo
*P. maldivensis*	cl, fl, en*	no	no	</>	r	b	ci-el	eq	ro	hi
*P. explanulata*	fl, en*	pars	no	>	r	c	ci-el	un	ro	hi
*P. minuta*	fl, en	pars	no	>	r-f	a	ci-el-in	eq	ro	lo
*P. xarifae*	en*	no	no	>	f	a	ci-el-in	un	ro	lo
*P. varians*	en*	pars	yes	<	f	a	ir-in	un	ro-fl	lo
*P. chiriquiensis*	en	pars	yes	<	f	a	ir-in	un	fl	lo
*P. giannii* sp. nov.	en	pars	no	<	f	b	po-ir-in	un	fl	lo

Based on growth form, *Pavona giannii* sp. nov. is distinct from the club-like *Pavona clavus* (Dana, 1846) and *Pavona maldivensis* (Gardiner, 1905), both also occasionally having free corallum margins. In these two species septa plunge from the corallite wall margin into the fossa (Fig. 8E, F), while in *P. giannii* sp. nov. septal upper margin within the calice is flattened and parallel to the corallum surface. Moreover, *P. maldivensis*’ corallites have typically raised walls (Fig. 8F) while corallites in the new species described herein are flush with the corallum surface, their wall margins never raised above it (Figs 2–5).

**Figure 9.**
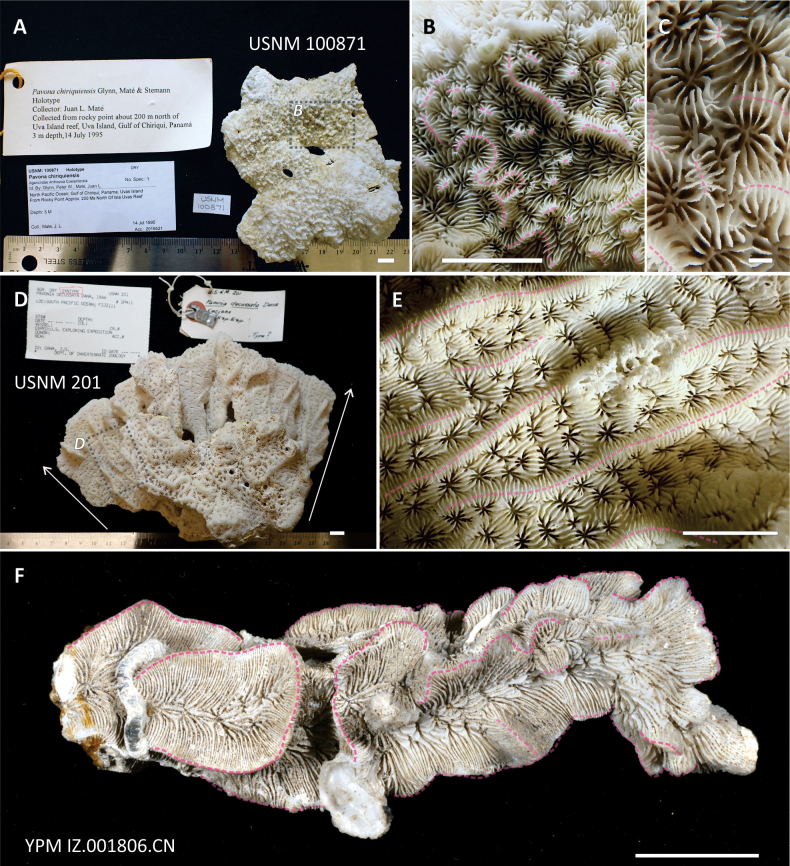
Type specimens of *Pavona chiriquiensis*, *Pavona decussata* and *Pavona varians* showing the position, arrangement, and length of the corallum ridges, their upper margin marked by transparent dashed pink lines. **A.** Top view of specimen USNM 100871, holotype of *P. chiriquiensis* from Uva Island, Panama; the transparent dashed grey polygon indicates the position of the surface portion shown in **B; B.** Detail of **A** showing irregularly arranged and shaped ridges on the corallum, some more elongated and others reduced to conical structures defined by different authors as “hydnophorae” (Glynn et al. 2001), or “conical monticules” (Veron and Pichon 1980); **C.** Close up view of the corallites and ridges of *P. chiriquiensis* paratype (YPM 24153); **D.** Side view of the anastomosing fronds in specimen USNM 201, syntype of *P. decussata* from Fiji, the two white arrows on the sides of it show the original colony growth direction from the base of the corallum to the fronds top margin; the transparent dashed grey polygon indicates the position of the surface portion in **B; E.** Detail of **C** showing long and well-defined, ridges growing along the frond and separating adjacent corallite series, the ladder-like arrangement of the radial elements running over the ridges is visible; **F.** View of specimen YPM IZ.001806.CN, syntype of *P. varians*, with well-developed crest-like ridges separating corallite series. Scale bars: 1 cm (**A, B, D–F**); 5 mm (**C**).

*Pavona cactus* (Fig. 8A), *Pavona decussata* (Dana, 1846) (Fig. 9D), *Pavona danai* (Milne Edwards, 1860), *Pavona divaricata* (Lamarck, 1816), *Pavona minor* (Brüggemann, 1879) and *Pavona frondifera* (Lamarck, 1816) form tall, bifacial and often anastomosing fronds and are therefore distinct from the encrusting *P. giannii* sp. nov. (Table 2). In these species, ridges form on the surface of a primarily encrusting corallum. These further develop into tall crests and eventually become fully formed bifacial fronds that can anastomose (Matthai 1948a, 1948b, Veron and Pichon 1980). Corallites are found on both lateral sides of these fronds, and additional ridges can eventually form like in *P. decussata* with the potential to further develop into more fronds. Therefore, even early-development stage colonies of these species are distinct from *P. giannii* sp. nov., typically devoid of ridges. Moreover, corallites in *P. cactus*, *P. danai*, *P. frondifera* and *P. minor* are smaller than 2 mm in largest diameter (Table 2) while *P. giannii* sp. nov. forms corallites wider than 2 mm in diameter.

Among the *Pavona* species forming predominantly encrusting coralla devoid of ridges, both *Pavona minuta* Wells, 1954 (Fig. 8G) and *Pavona xarifae* Scheer & Pillai, 1974 form corallites less than 2 mm in diameter, hence smaller than in the new species, while *P. explanulata* (Lamarck, 1816) (Fig. 8H) has corallites more than 4 mm in diameter, larger than in the new species. Furthermore, in these three species, corallites can be more than 1 corallite diameter apart while they are always less than one corallite apart in *P. giannii* sp. nov.

The two encrusting *Pavona* species that bear more similarity at the corallite level to the new species described here are *Pavona chiriquiensis* Glynn, Maté & Stemann, 2001 and *Pavona varians* (Verrill, 1864) (Table 2). However, both species have smaller corallite diameter (Table 1) based on the morphometric analyses performed by Maté (2003). Moreover, *Pavona chiriquiensis* has S1 = S2 > S3 and S3 are ornamented with same shape and size granules as S1–2 (Glynn et al. 2001: fig. 2B), while *Pavona giannii* sp. nov. has smooth S3 (Fig. 5G–L). While the new species and *P. varians* have a similar minimum corallite diameter, corallites in the latter are on average 1 mm smaller in maximum diameter than in *P. giannii* sp. nov. corallites. Consequently, *P. varians* has shorter S1 radial elements and has on average less septa per corallite, but more of them reach the columella (Table 1). Finally, both *P. varians* and *P. chiriquiensis* form variably long ridges that never develop into fronds (Fig. 9A–C, F). In *P. varians*, ridges can be straight or contorted, develop radially or perpendicularly to the colony margin (Lewis et al. 2025: fig. 2), and in larger colonies their distribution and development become more irregular (Matthai 1948b). Because of such variability, these structures in the genus *Pavona* have been referred to with different terms, namely “crests” (Verrill 1864), “vertical keels” (Gardiner 1898), “ridges” (Matthai 1948a, 1948b; Veron and Pichon 1980; Latypov 2014; Lewis et al. 2025), “collines” (Matthai 1948a, b; Veron and Pichon 1980; Sheppard and Sheppard 1991; Glynn et al. 2001; Maté 2003; Latypov 2014), for the more elongated variants, and “monticules” (Matthai 1948a), “conical monticules” (Veron and Pichon 1980), and “hydnophorae” (Glynn et al. 2001) for the shorter ones. Regardless of the length or orientation, radial elements run over the raised ridges in *Pavona* species perpendicularly to their main axis leading to a typical ladder-like arrangement (dashed pink lines in Fig. 9). This becomes less obvious when a ridge further develops in height into a crest or a frond. Although *P. giannii* sp. nov. is devoid of ridges on the corallum surface, more or less elongated walls enclosing series of corallites can form. Their position is detectable because the strongly alternating septa are neatly and regularly arranged perpendicular to their direction and form the same ladder-like arrangement (dashed pink lines in Figs 2, 3, 5) observed over ridges in species where these form.

Historically, polyp features in scleractinian corals are seldom considered in taxonomic descriptions especially for taxa described before sampling and observations could take place during SCUBA diving or in aquarium facilities. Some notable exceptions are represented by genera like *Euphyllia* Dana, 1846 and *Fimbriaphyllia* Veron & Pichon, 1980 in which polyp shape and coloration bear more informative characters than the actual skeleton (Veron and Pichon 1980; Luzon et al. 2017; Arrigoni et al. 2023). In taxa like *Blastomussa* Wells, 1968, *Alveopora* Blainville, 1830, *Goniopora* de Blainville, 1830, *Bernardpora* Kitano & Fukami, 2014 and *Porites* Link, 1807 polyp characters have been shown to be informative or diagnostic (Benzoni et al. 2014; Kitano et al. 2014; Terraneo et al. 2016, 2019). In most Agariciidae animal features in vivo are barely visible due to the small size and transparent nature of the polyps (Veron and Pichon 1980). Nevertheless, polyp expansion in the daytime and pale coloration of the tentacles are typical and diagnostic features of *P. giannii* sp. nov. throughout its known geographic distribution range regardless of the local environmental conditions. Maté (2003: 431) remarked that in the Pacific coast of Panama in his study of *P. frondifera*, *P. chiriquiensis* and *P. varians* “none of the three *Pavona* species displays expanded polyps during the day except during periods of rapid water flow”. Glynn et al. (2001) and Maté (2003) indicate that tissue coloration in vivo is a diagnostic character to identify *P. chiriquiensis* in the field and to distinguish it from congeners. Maté (2003: 438) remarked that “dark polyps and coenosarc, and contrasting bright white to silvery oral discs and tentacles” characterized the *P. chiriquiensis* colonies in Panama. This character seems to be conserved also at other locations. In fact, the same coloration was consistently observed during the coral biodiversity surveys in the Marquises Archipelago, French Polynesia, where *P. chiriquiensis* was the most frequently encountered scleractinian coral found at 94% of the surveyed sites (Salvat et al. 2016: fig. 4A). Amongst congeners, *P. gigantea* and *P. explanulata* (Fig. 7C) usually have polyp tentacles variably extended during the daytime, however they are grey to white in the former and green to brown in the latter, and both species have different growth forms and polyp/corallite size (Table 1). Finally, the in vivo coloration of *P. distincta* is unknown (Latypov 2014).

## ﻿Conclusions

*Pavona giannii* sp. nov., a new species of colonial hard coral in the genus *Pavona*, is described, highlighting the diagnostic value of both skeletal and polyp morphology. This species, with its distinctive encrusting growth form, smooth corallum surface, and characteristic daytime polyp expansion and coloration, stands apart from all currently recognized *Pavona* species. It inhabits shallow water coral reefs and hard grounds in the Indian Ocean. A detailed comparative analysis, including morphometric data and comparisons with type and reference material, underscored the morphological distinctiveness of *P. giannii* sp. nov. and provided an illustrated summary of the morphological variability within the genus. Future research on *Pavona* and other Agariciidae, should prioritize expanded species sampling from diverse geographic localities including type localities, coupled with phylogenomic analyses. Such efforts will be essential to resolve the complex phylogenetic relationships within *Pavona* and ultimately establish a robust and stable taxonomic framework for this ecologically important coral genus.

## Supplementary Material

XML Treatment for Pavona giannii

## ﻿Additional information

### Use of AI

No use of AI was reported.

### Author ORCIDs

Francesca Benzoni https://orcid.org/0000-0001-8906-1309

### Data availability

All of the data that support the findings of this study are available in the main text.
